# Observation of a slanted moisture structure with weak updraft leading to localized heavy rainfalls

**DOI:** 10.1038/s41598-025-02314-2

**Published:** 2025-07-02

**Authors:** Satoru Yoshida, Tetsu Sakai, Tomohiro Nagai, Hiromu Seko, Teruyuki Kato, Koichi Shiraishi, Shingo Shimizu

**Affiliations:** 1 Meteorological Research Institute, Tsukuba, Japan; 2 Fukuoka University, Fukuoka, Japan; 3 National Research Institute for Earth Science and Disaster Resilience, Tsukuba, Japan

**Keywords:** Atmospheric dynamics, Natural hazards

## Abstract

Understanding moisture structures within moist low-level jets (MLLJs) that fuel moisture into mesoscale convective systems (MCSs) is essential for revealing the development of MCSs that cause localized heavy rainfalls. The moisture structure in MLLJs and the mechanisms that determine it are unclear. We used radiosondes, a water vapor lidar, and Doppler lidar to observe MLLJs in Kyushu, western Japan. The observations revealed an unstable moist layer that was saturated or nearly saturated and that extended vertically from near the surface to more than 1 km above sea level in the MLLJ. The thickness of the moist layer gradually increased with a slant angle of less than 1° toward the MCS. The MLLJ involved weak updrafts below the cloud bases that contributed to thickening the slanted moist layer via vertical transport of moisture. The slanted moisture structure with a weak updraft appeared to effectively fuel moisture into MCSs and led to localized heavy rainfalls.

**Supplementary Information:**

The online version contains supplementary material available at 10.1038/s41598-025-02314-2.

## Introduction

Mesoscale convective systems (MCSs), which are deep and organized convective storms, develop in many regions, including the USA, Europe, and East Asia^1–3^, and they often produce localized heavy rainfalls that lead to floods. Previous studies have documented increases of intense rainfall caused by MCSs and have suggested that such intense rainfall from MCSs is likely to continue increasing in the future warmed climate^4–8^. Understanding the mechanisms that create MCSs and improving the skill of forecasting the localized heavy precipitation associated with MCSs are therefore essential.

A common characteristic across various types of MCSs is the presence of a moist low-level jet (MLLJ)^9^. Because the latent heat of the moisture in MLLJs is the primary driver of the development of convective clouds that form MCSs, the moisture structure in MLLJs is highly related to the development of MCSs^10–16^, but the relationship between them dose not seem to be simple^17^. Numerical simulations indicated that a difference of only 1 g k^−1^ in boundary layer moisture could determine whether convection fails to initiate or develops^18^. Semi-idealized numerical simulations showed that a small moisture increase of 2 g kg^−1^ in the low-levels (a 3.4% increase in vertically integrated moisture) produced a 60% increase in area-integrated precipitation and altered the locations of the heavy rainfall region^17^. These results indicate that where MCSs initiate, how they develop, and how much precipitation they produce are highly sensitive to the moisture structure in the MLLJ of the MCS. To understand the mechanisms responsible for the formation of MCSs, detailed moisture structures in MLLJs and their formation mechanisms are essential. However, the structure of moisture and its formation mechanism within MLLJs leading to heavy precipitations remains poorly understood, since detailed moisture observation in the MLLJ is insufficient. Therefore, clarifying the actual conditions and its development mechanism through observations are needed to improve numerical simulations for heavy precipitation associated with MCSs.

In the East Asia, a stationary front often develops, resulting in heavy rainfall across Taiwan, mainland China, the Korean Peninsula, and Japan^19–21^. The stationary front typically forms around May near Taiwan and/or the southeastern coasts of mainland China, gradually moving northward. By late June to early July, it reaches Kyushu, western Japan, marking the onset of the rainy season in Kyushu. During the rainy season in Kyushu, MLLJs form over the ocean along the periphery of the North Pacific High, transporting substantial amounts of moisture since it receives moisture from warm sea surface. The MLLJ in this region exhibits distinct differences from the nocturnal MLLJ frequently observed over the mid-continent of the United States. In contrast to the MLLJ over the Great Plains, which is typically associated with stable layers near the surface^11,22,23^, the MLLJ in Kyushu often lacks a stable layer in the lower atmosphere during nighttime and morning^24–26^. This unique characteristic sometimes allows MCSs to develop in very wet conditions. Moisture structures in MLLJs around Kyushu, where many MCSs occur^27^, have been studied via numerical simulations^28–31^ and objective data analyses^32–34^. A numerical simulation of an MCS producing heavy rainfall in Kyushu has reproduced a moist layer extending vertically from the surface to 850-hPa level^29^. Objective data analyses of an MCS event in Kyushu have shown that the altitudes of equivalent potential temperature (${\theta }_{e}$) surface within the MLLJ increase toward the MCS^32^.

A few observations of the moisture structures of MCSs around Kyushu have also been conducted^12,24–26,35–37^. Water vapor Raman lidar (RL) observations have revealed a moist layer extending up to 1.6 km above sea level (asl) in a MLLJ^36^. Although these observations have revealed details of the vertical moisture structures and characteristics of the moisture in MLLJs during the rainy season in Kyushu, no observation has revealed the three-dimensional (3D) moisture structures within MLLJs associated with MCSs. In addition, while upward development of the moist layer in a MLLJ has been discussed^36^, no observation has revealed vertical moisture transport in a MLLJ in this region. The lack of such important observations has hindered research on the localized heavy rainfall caused by MCSs during the extremely humid rainy season in East Asia.

**Fig. 1:**
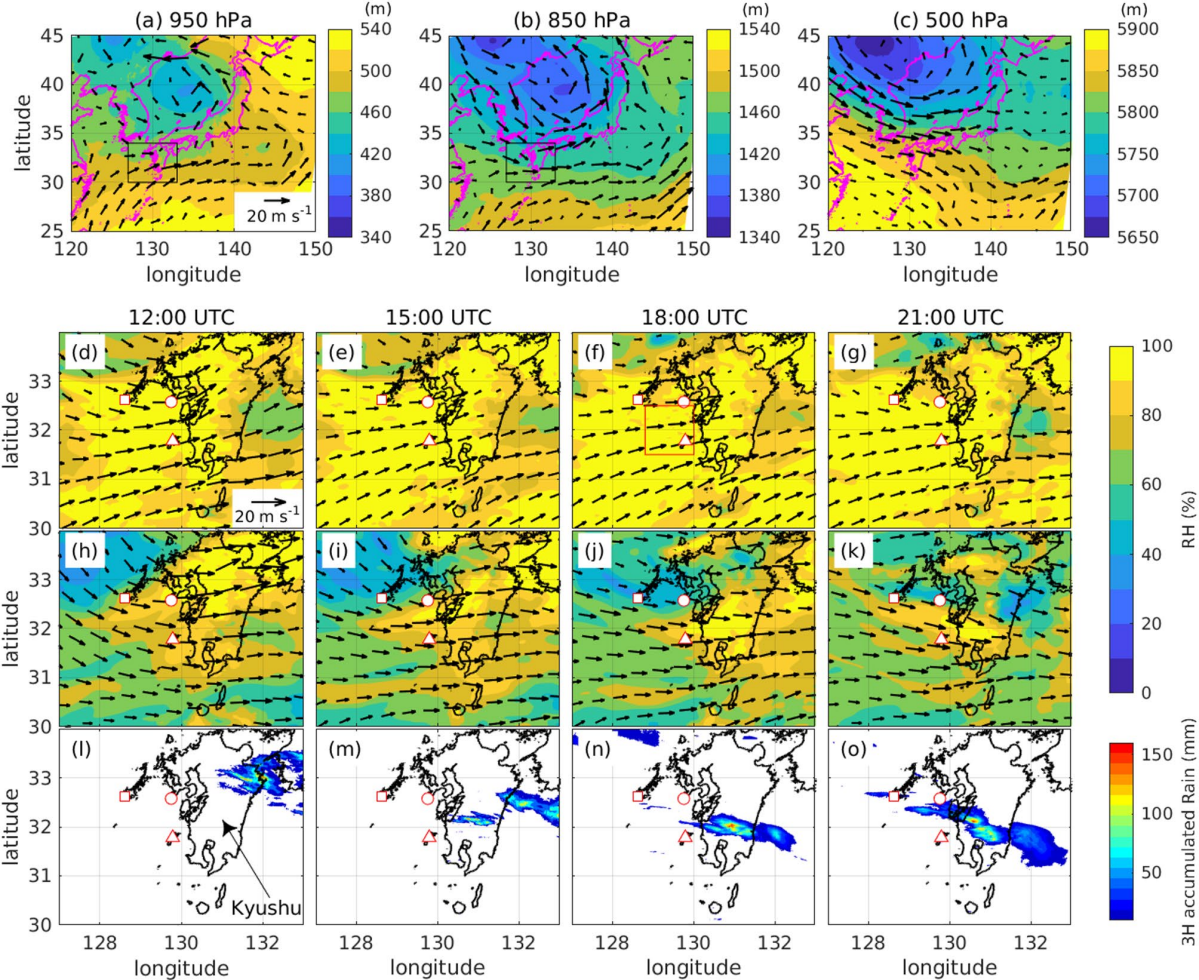
An overview of the event from the local analysis. (**a**–**c**) represent the geopotential height (color) and horizontal wind (arrows) at 15 UTC on 14 July 2022 at pressure levels of (a) 950 hPa, (**b**) 850 hPa, and (**c**) 500 hPa. (**d**–**g**) represent relative humidity (RH) fields and horizontal wind (arrows) at 950 hPa level at (**d**) 12:00 UTC, (**e**) 15:00 UTC, (**f**) 18:00 UTC, and (g) 21:00 UTC. (**h**–**k**) are the same as (**d**–**g**), but at the 850 hPa level. (**l**–**o**) represent accumulated three-hour precipitation from the weather radar and rain gauge analysis from (l) 12:00 UTC, (**m**) 15:00 UTC, (**n**) 18:00 UTC, and (**o**) 21:00 UTC. The black rectangles in (a) and (b) represent the domains in (**d**)–(**o**). Red squares, circles, and triangles filled with white represent the locations of the Fukue, Nomozaki, and Koshiki sites, respectively. The red rectangle in (f) indicates the region where updraft associated with low-level convergence and Ekman pumping were calculated. Reference arrows of horizontal wind for (a) through (c) and (d) through (k) are shown in (a) and (d), respectively. The location of Kyushu is indicated in (l).

To clarify the 3D moisture structure of an MLLJ associated with an MCS, we conducted an observation campaign in Kyushu. We made observations at three sites: Fukue Island (Fukue site), Nomozaki (Nomozaki site), and Shimokoshiki Island (Koshiki site) (Fig. 1d–o). We observed vertical profiles of moisture and wind using RL and Doppler lidar (DL) at the Koshiki site, and we launched radiosondes every three hours from all three sites. The vertical profiles obtained by radiosondes released at nearly the same time from the three observation sites revealed the 3D moisture structure in the MLLJ associated with an MCS on 14 July 2022. This paper describes the observation results and discusses the mechanisms responsible for the formation of this moisture structure.

## Results

### Overview of the event

On 14 July 2022, low pressure systems were detected over the Sea of Japan and mainland China (Fig. 1a–c). To the south of these systems, southwesterly to westerly winds below the 850-hPa level were evident around Kyushu and the East China Sea (around the rectangular areas in Fig. 1a,b). While regions of relative humidity (RH) greater than 80% at the 950-hPa level persisted around Kyushu (Fig. 1d–g), those at 850 hPa and associated precipitation areas in Kyushu moved southward from 12 to 18 UTC (Fig. 1h–o). Around 17 UTC, numerous convective clouds that occurred over the southern part of Kyushu formed an MCS (Figs. S1 and S2). As seen in Fig. S2, most convective clouds (labeled ‘b’ thorough ‘h’) were initiated on the upwind (west) side of the pre-existing convective clouds and propagated along a similar path to that of the pre-existing convective clouds. For example, around 17:14 UTC, convective cloud ‘a’ developed eastward, while convective cloud ‘b’ was newly initiated on the windward side of the existing convective clouds and propagated eastwards. This feature suggests that this MCS is back-building type^38^. The six-hour cumulative precipitation associated with the MCS from 18 UTC on July 14 exceeded 320 mm in the southern part of Kyushu. In this study, we focused on the moisture structure within the MLLJ, which was situated on the upwind side of the MCS and passed over the three observation sites.

In this event, we did not find either a synoptic-scale front or a mesoscale front associated with the MCS. In addition, a mesoscale vortex at 500 hPa above mainland China was far enough away that it did not affect the MCS (Fig. 1c). It appears that the MCS developed without fronts or a mesoscale vortex.

## Radiosonde observations

**Fig. 2:**
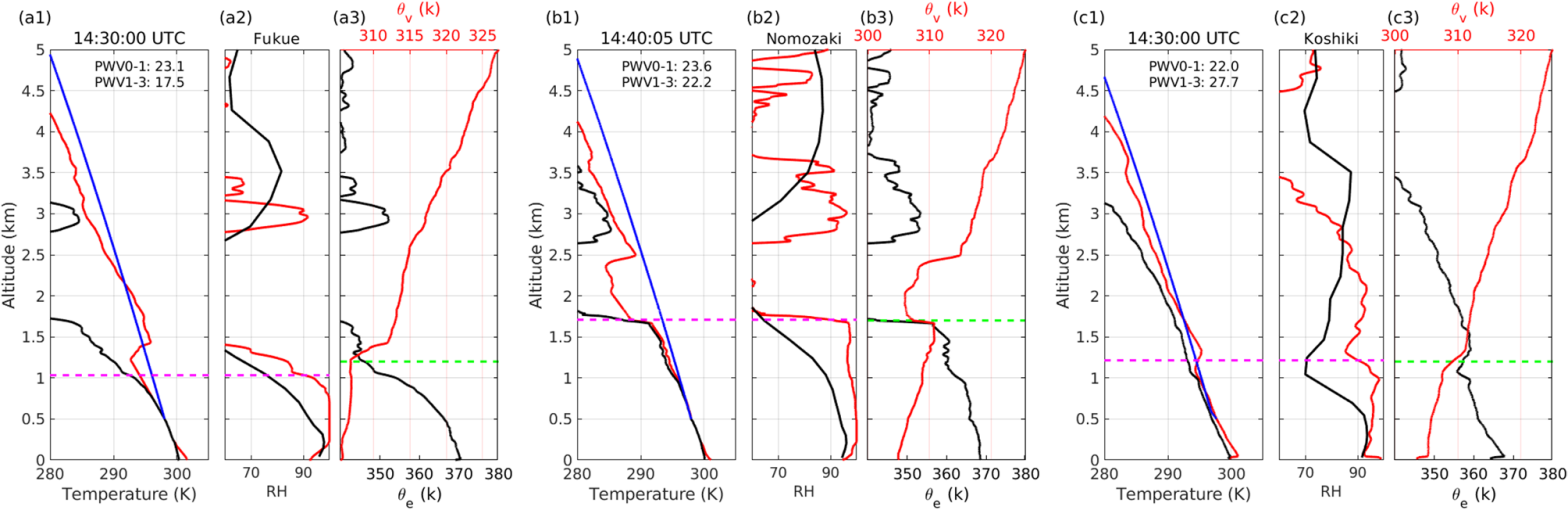
Observation results of radiosondes released from the three sites at approximately 14:30 UTC on 14 July 2022. (a) observations of radiosonde released from the Fukue site. (**a1**) vertical profiles of temperature (red line), dewpoint (black line), and temperature of a parcel released at 500 m asl (blue line). (**a2**) vertical profiles of RH from radiosonde observations (red line) and RH interpolated at the Fukue site from LA data at about 30 min after the radiosonde release (black line). (**a3**) equivalent potential temperature (${\theta }_{e},$ black line and bottom axis) and virtual potential temperature (${\theta }_{v},$ red line and top axis). The radiosonde release time is shown at the top of (a1). Magenta horizontal dashed lines in (a1) and (a2) represent RH90H. The green horizontal dashed line in (a3) represents the convective mixing layer height. Precipitable water vapor (PWV) calculated from radiosonde observations are shown in (a1). PWV0-1 and PWV1-3 show PWV in kg m^−2^ between the surface and 1 km asl, and between 1 and 3 km, respectively. (b) is the same as (a) but for a release at 14:40:05 from the Nomozaki site. (**c**) is the same as (**a**) but for a release at 14:30:00 from the Koshiki site. All radiosonde results are provided in Fig. S3.

During the event, low-level moist layers (> 90% RH) were observed by the radiosondes in the MLLJ at all observation sites at around 14:30 UTC (Fig. 2). To examine the depth of the moist layer, we defined RH90H as the altitude below which all RHs above 10 m asl exceeded 90% (horizontal magenta dashed lines in Fig. 2). The threshold value of 90% in RH90H was set because most RH profiles exhibit significant changes around 90%, and this value effectively distinguishes the lower moist layer from the upper relative dry layer (Fig. S3). The fact that the derivatives of ${\theta }_{e}$ with respect to altitude ($\frac{\partial {\theta }_{e}}{\partial z}$) were mostly negative below the RH90Hs was indicative of potential instability. We calculated temperature profiles of parcels released at 500 m asl (blue lines in Fig. 2). It is apparent in Fig. 2 that the parcels reached their levels of free convection (LFC) after only a small ascent or almost no ascent. Because the temperature of the environment was very close to the temperature of the parcels below the RH90Hs, parcels below the RH90H would have weak buoyancy above the LFC and receive very small energy from the environment. Indeed, the convective available potential energies below RH90H (CAPE_RH90Hs), defined in this paper as the integration of the upward buoyancy force from the LFC for a parcel released at 500 m asl to RH90H, were 20, 32, and 4.4 J kg^−1^, respectively, at Fukue, Nomozaki, and Koshiki sites at around 14:30 UTC (Fig. 2). These CAPE_RH90Hs are relatively small for a severe convective environment to initiate deep convection. Note that CAPE_RH90H and convective available potential energy (CAPE) are the same in that they both integrate buoyancy in the vertical direction. However, while the CAPE is integrated from LFC to equilibrium level, CAPE_RH90H is integrated from LFC to RH90H. We introduced CAPE_RH90H to quantify the buoyancy force gained by a parcel below RH90H from the environment.

We observed moist absolute unstable layers (MAULs), which are saturated with water vapor and unstable and play an important role in the development of MCSs^39^. The MAUL is determined in this paper if the radiosonde observation results satisfy the following both Eqs. (1) and (2) described in^39^.

1 $$T-{T}_{d}<1$$

2 $$\frac{\partial {\theta }_{e}}{\partial z}<0$$

$T$ and ${T}_{d}$ represent the temperature and dewpoint temperature. Below RH90Hs, ${\theta }_{e}$ typically decreases with altitude, so its altitude derivative is considered negative when evaluating MAUL conditions. In Fig. 2, air masses that satisfied the MAUL criterion (Eqs. 1 and 2) were between 63 and 993 m at the Fukue site, below 1.69 km at the Nomozaki site, and between 190 m and 1.14 km at the Koshiki site. Around 11:30 UTC the air masses that satisfied the MAUL criterion were between 69 m and 1.17 km at the Fukue site, between 46 m and 2.18 km at the Nomozaki site, and between 891 m and 1.05 km at the Koshiki site (Fig. S3b,g,l). These results indicated the existence of shallow MAULs with thicknesses of less than 2.2 km at the observation sites.

To examine the horizontal structure of the moist layer, we compared the RH90Hs at the Fukue and Nomozaki sites. Since the Fukue site was located on the upwind side (west) of the Nomozaki site, comparisons of the results of the observations at Fukue to those at Nomozaki revealed the horizontal structure of the moist layer from the upwind side to the downwind side. Around 14:30 UTC, the RH90H was deeper at Nomozaki (1.7 km) than at Fukue (1.0 km). An implication was that the moist layer in the MLLJ had a slanted structure that gradually thickened toward the MCS. The slant angle of the upper boundary of the moist layer was 0.36°. We also manually estimated the height of convective mixing layer (CML) in each radiosonde observation, based primarily on the vertical profiles of virtual potential temperature (${\theta }_{v}$) and RH (Fig. 2). The RH90Hs and heights of CML were similar in almost all radiosonde observations. The CML therefore included the MAUL or close to the MAUL condition and was gradually thickening toward the MCS. Similar results were obtained around 11:30 UTC (Fig. S3b,g). The RH90H was deeper at Nomozaki (2.2 km) than at Fukue (1.2 km), and the slant angle of the upper boundary of the moist layer was 0.55°. Since moist layers exceeding 1 km and shallow MAULs were observed almost simultaneously at both the Koshiki and Nomozaki sites (Fig. 2b,c), the moist layer extended horizontally more than 80 km, the distance between the Nomozaki and Koshiki sites, perpendicular to the MLLJ flow. The wide MLLJ with the slanted rise of moist layer implied that the MCS developed through layer lifting in which a wide moist layer ascended slantwise^40^.

Note that the RH was lower in the local analysis (LA) data than in the radiosonde observations in most cases below the RH90H (Fig. S3). These differences made it difficult to discuss the low-level moisture structure using LA data in this case. Generally, output of mesoscale models with a horizontal grid interval of 5 km or more, including LA, are sometimes insufficient to simulate moisture in CMLs^41,42^.

### RL and DL observations

**Fig. 3:**
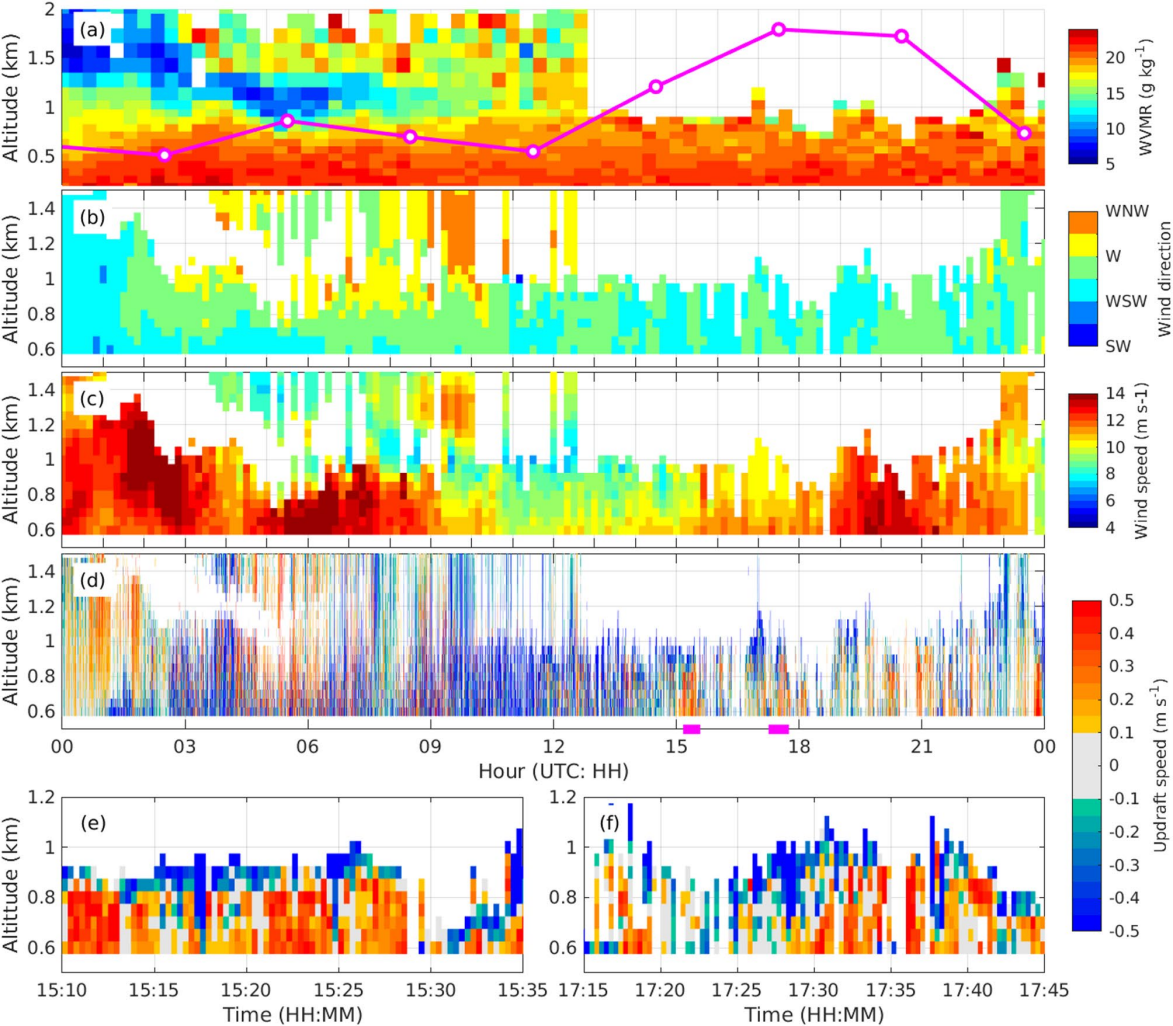
Time-height plot of the RL and DL data at the Koshiki site on 14 July 2022. (**a**) time-height plot of WMVR obtained with the RL at Koshiki (color plots). The magenta line represents the RH90H observed by the radiosondes launched from the Koshiki site. (**b**) and (**c**) show 10-min averaged horizontal wind direction and speed, and (**d**) shows the vertical wind velocity every 19 s observed by the DL at the Koshiki site. (**e**) and (**f**) are magnified views of (**d**) at the magenta horizontal lines in (**d**). The positive and negative values in (**d**)–(**f**) represent updraft and downdraft, respectively.

Because of the presence of cloud bases at approximately 800 m asl above the Koshiki site after 12 UTC July 14, the RL and DL data were limited below ~ 800 m asl. The water vapor mixing ratio (WVMR) around 500 m asl remained quite high throughout the day, and it showed no distinct increase between 12 and 20 UTC (Fig. 3a). The horizontal wind speed at 700 m asl observed by the DL gradually increased from 9.2 m s^−1^ at 12 UTC to 13.5 m s^−1^ at 20 UTC (Fig. 3c), and the direction of the wind remained from west-southwesterly to westerly (Fig. 3b). The increase of the horizontal wind speed intensified the moisture flux into the MCS and contributed to its development. Because the slanted moist layer, confirmed by the radiosonde observations, moved southward (Fig. 1h–j), the MLLJ over the Koshiki site had a slanted moisture structure toward the MCS.

During the onset of the MLLJ when the RH90H at the Koshiki site increased between 14:30 and 20:30 UTC, several intervals of weak updrafts lasting several minutes were observed (Fig. 3d). Figure 3e,f shows that most of the vertical velocities below the cloud bases were positive between 15:18 and 15:24 UTC (17:28 and 17:42 UTC). The mean updraft using only positive values (the mean horizontal velocity) at 700 m asl between 14:30 and 20:30 UTC was 0.17 m s^−1^ (11.3 m s^−1^). The angle formed by the average updraft (0.17 m s^−1^) and average horizontal velocity (11.3 m s^−1^) was 0.86°, comparable to the slant angles of the upper boundary of the slanted moist layer obtained from the radiosonde observations (0.36° and 0.55°). These weak updrafts helped extend the moist layer vertically and contributed to the formation of the slanted moisture structure in the MLLJ. The average moisture density at 717 m asl between 14:30 and 20:30 UTC was 21.0 g m^−3^ based on the RL and radiosonde data. If the average vertical velocity in the updraft region below the cloud bases was assumed to be 0.17 m s^−1^, the estimated average rate of the amount of moisture transported upward into the cloud bases was 3.58 g m^−2^ s^−1^.

Note that most vertical velocities around the cloud bases (~ 800 m asl or higher) in Fig. 3e,f are negative, and the reason is unclear. We hypothesize that the negative values were caused by complex processes such as water vapor condensation, cloud particle evaporation, and cloud particle growth.

## Discussion

### Characteristics of MAULs and their roles for localized heavy rainfall

Layer lifting with strong convergence sometimes enables MAULs to form on the upwind side of thunderstorms^39^. This scenario has been supported by previous studies^32,33^. The thicknesses of the MAULs with strong convergence appear to be more than a couple of kilometers^32,33^. However, several studies have shown that shallow MAULs can form without strong convergence^13–15^. Examination of radiosonde data on the upwind side of MCSs has revealed formation of shallow MAULs with depths of less than 50 hPa above nocturnal stable layers in weak updraft regions^14^. A composite analysis of radiosonde observations has shown saturated layers with depths of less than 100 hPa; an implication is that shallow MAULs can be present above a nocturnal stable layer^13^. A shallow MAUL with a depth of 30 hPa above a nocturnal stable layer has been reproduced in a numerical simulation^15^. The MAUL observed in this study was also a shallow MAUL; its thickness was less than 2.2 km (Fig. S3), and it was located on the upwind side of the MCS. These characteristics were similar to those of the shallow MAULs reported in previous studies^13–15^, but the MAULs in this study formed without a stable layer.

Analysis of the Japan Meteorological Agency operational Mesoscale Model^43^ has shown the existence of a slanted moist structure with deep MAULs that was likely related to strong convection because it was co-located with areas of precipitation^32^. In contrast, we observed a slanted moist structure involving a shallow MAUL without strong convection. The results of previous studies^13–15,32,33,39^, in conjunction with this study, have suggested that there are two types of MAULs. One type occurs away from convective regions and is associated with weak updrafts, a shallow depth^13–15^, and a small slant angle of moist layer. The MAUL in this study fell into this first category. The other type occurs near or in almost the same location as strong convection associated with strong convergences, and it has a greater depth^32,33^. In the first type, shallow MAULs form and develop slowly with weak updrafts because they are distant from convection. In the second type, large dynamical forces produce relatively deep MAULs near strong convection.

In the case of layer lifting with a warm, moist layer containing MAULs, it has been suggested that the upper part of the layer is extensively heated by sensible heat released from the moist air rising from the lower layer^39^, and consequently the temperature difference between the rising layer and the environment becomes smaller. That smaller temperature difference decreases the parcel’s acceleration associated with buoyancy in the MAULs. In this study, we observed the very weak updrafts in the slanted moist layer that had a shallow MAUL with the small CAPE_RH90H. Our observations clearly demonstrated the characteristics of MAULs that have previously been suggested^39^. They implied that the MAUL could effectively transport large amounts of moisture into the MCS and contribute to the heavy precipitation, since the weak updrafts derived by the weak buoyancy in the MAUL favor maintaining the MAUL^39^. It seems that the MAUL in this studied case transported a large amount of moisture into the MCS and contributed to its development while maintaining its state.

### Vertical moisture transport in the MLLJ

The results of the observations indicated that the average rate of upward moisture transport into the cloud bases in the MLLJ was 3.58 g m^−2^ s^−1^. Since the vertical pressure velocities below 750 hPa during 11:30–17:30 UTC were mostly negative (Fig. S4), we hypothesize that the moisture in the region of the weak updraft below the cloud bases was transported upward into the 750-hPa level and that the weak updraft persisted for several hours. If the updraft persisted for one hour, the total vertical moisture transported for one hour was 12.9 kg m^−2^ at 717 m asl. It is likely that some of the vertically elevated moisture might have condensed, but some likely increased the RH in the upper layers and elevated the height of CML. As is apparent in Fig. S3k,n, precipitable water vapor (PWV) below 1 km increased by 9.5% (1.9 kg m^−2^) from 08:30 UTC (before the onset of the MLLJ) to 17:30 UTC (the onset of the MLLJ), and PWV between 1 and 3 km concurrently increased by 15.9% (3.7 kg m^−2^). An implication is that the increase of the moisture content of the atmosphere was greater between 1 and 3 km asl than below 1 km during the onset of the MLLJ. The increase in PWV at altitudes of 1–3 km can be explained by the prolonged upward transport of moisture below the cloud base by weak updrafts. Since the atmosphere below 1 km was relatively humid, these weak and moisture-rich updrafts had small effect on PWV at that level. However, they caused a larger increase in PWV between 1 and 3 km asl, where the air was initially drier than the lower altitudes.

Numerical simulations and objective data analyses indicated that the heavy precipitation associated with the MCSs was caused by moist conditions not only at low-levels but also at mid-levels (approximately the 900-hPa level or higher)^15,32,44^. Our observations also revealed an increase of moisture at middle levels (between 1 and 3 km asl). Such observational features are consistent with results^15,32,44^. In addition, our results suggested that vertical moisture transport below the cloud bases could be sufficient to moisten the mid-level. Numerical simulations and objective data analysis have revealed increases of moisture at mid-levels (about 2 km asl or at 850 hPa) before the onset of MCSs and have indicated that the increases of moisture are caused by horizontal transport of moisture^15,37^. This study, in conjunction with their results, indicates that both horizontal and vertical transport of moisture could contribute to the increase in mid-level moisture.

Previous studies^36,45^ have suggested that weak updrafts in MLLJs are partially intensified by buoyancy associated with increases of moisture. However, the RL observations showed no clear increase of WVMR around 500 m asl at the Koshiki site during the onset of the MLLJ. Effects due to buoyancy related to moisture increases were therefore limited in this event. Low-level convergence, isentropic ascent^13^, and Ekman pumping^31^ have been suggested as possible drivers of low-level weak updrafts in the weak lifting environment on the upwind side of MCSs. We used LA data to examine these three candidates in the current case. First, we estimated the updraft caused by low-level convergence. The average convergence at 18 UTC at the 950-hPa level around the Koshiki site, indicated by the red rectangle in Fig. 1f, was 2.8 × 10^−5^ s^−1^. We estimated that if this average convergence extended from the surface to 700 m asl, the updraft velocity related to the low-level convergence at 700 m asl would be 0.020 m s^−1^. Second, we estimated the updraft velocity caused by isentropic ascent. Figure S5a indicates that the potential temperature (θ) at 950 hPa rose toward the west (around 125E°) and was lower near the Koshiki site. This pattern was attributable to the west-to-east decrease of the sea surface temperature (SST). The magenta dashed line in Fig. S5b indicates the rise of the 306 K isentrope of θ from the upwind side into the MCS at a slant angle of 0.09°. The updraft speed of a parcel propagating on the 306 K isentropic surface can be estimated to be 0.016 m s^−1^ if the horizontal wind velocity is 10 m s^−1^. Finally, we estimated the contribution from Ekman pumping. The estimated updraft velocity at the top of the CML associated with Ekman pumping was equated to $\frac{H}{2\pi }\left(\frac{\partial {V}_{g}}{\partial x}-\frac{\partial {U}_{g}}{\partial y}\right)$, where $H$, ${U}_{g}$, and ${V}_{g}$ represent the height of CML and the zonal and meridional geostrophic winds at the top of the height of CML, respectively^46^. By assuming that the height of CML was at 850 hPa and the geostrophic wind speed at 850 hPa is approximated by the horizontal wind speed, we were able to estimate the maximum updraft associated with Ekman pumping in the red rectangle at 850-hPa level in Fig. 1f to be 0.040 m s^−1^. None of the three mechanisms (low-level convergence, isentropic ascent, and Ekman pumping) alone can fully explain the weak updrafts (0.17 m s^−1^) observed by the DL. It appears that all three mechanisms worked together to generate the weak updrafts at low-levels and thicken the moist layer in the CML. The estimation of the updraft in this study was conducted using objective analysis data, which may not fully reflect reality. Therefore, future research needs to clarify the mechanisms that causes this updraft through observations.

## Summary

In this study, we conducted an observational campaign using the RL, DL, and frequent radiosonde releases in Kyushu, western Japan, to investigate the moisture structure within the MLLJ on the upwind side of an MCS. Comparisons of radiosondes released almost simultaneously from three observation sites revealed a moist structure slanted from the upwind side toward the MCS. The slant angle of the upper boundaries of the moist layer was less than 1°. Since the radiosonde observations indicated that the heights of CML and RH90Hs were similar, it appears that the CML, filled with MAUL or close to MAUL, was gradually thickening as it moved toward the MCS. The slanted moist structure contained updraft regions, but the updrafts were weak, and their average velocity was 0.17 m s^−1^. These weak updrafts may have played a key role in moistening the mid-level atmosphere by transporting moisture vertically and thickening the slanted moist layer. The slanted moisture structure with very weak updrafts might effectively transport large amounts of moisture into the MCS and lead to localized heavy rainfall in the MCS.

## Methods

On 14 July 2022, we launched radiosondes almost simultaneously from the three sites every three hours. We applied 5-point moving averages to the temperature and relative humidity profiles obtained from radiosonde data. We used the method described in a previous report^14^ to estimate the vertical pressure velocity (ω) from the radiosondes released from the three stations. We calculated the horizontal divergence (${\nabla }_{p}\cdot V$) in each 5-hPa level from 950 to 150 hPa.

3 $${\nabla }_{p}\cdot V=\frac{1}{A}\frac{dA}{dt}$$

where V and A represent the horizontal wind speed and area of a triangle with vertices at the three radiosonde locations at the same pressure level, respectively. We next adjusted the horizontal divergence to ensure that there was no overall horizontal divergence in the vertical column^47^. The adjusted horizontal divergences ($\overline{{\nabla }_{p}\cdot V}$) were calculated by subtracting $\frac{2D\left(i-1\right)}{N\left(N-1\right)}$ from the original horizontal divergence in Eq. (3). *D*, *N*, and *i* represent the vertically integrated original horizontal divergence, the number of pressure levels, and the pressure level index, respectively, where $i=1$ at the bottom and $i=N$ at the top. We then integrated the adjusted horizontal divergence from 950 hPa to the desired pressure level (p) to estimate $\omega \left(p\right)$,

4 $$\omega \left(p\right)=\omega \left({p}_{950}\right)-{\int }_{{p}_{950}}^{p}\overline{{\nabla }_{p}\cdot V}dp$$

where ${p}_{950}$ represents 950 hPa. In this paper, $\omega \left({p}_{950}\right)$ was assumed to be zero since most of the area enclosed by the three observation sites was above the sea, and the updrafts caused by terrain were limited. The $\omega \left(p\right)$ estimated in Eq. (4) represents the average vertical pressure velocity in a triangle with vertices at the three radiosondes.

We deployed the RL and DL (WindCube 200s, VAISALA) at the Koshiki sites. The RL at the Koshiki site emitted laser pulses vertically at a wavelength of 355 nm with average energy of 200 mJ and pulse repetition frequency of 10 Hz. It received Raman-scatted light signals at wavelengths of 407 nm (H_2_O Raman) and 387 nm (N_2_ Raman) as well as elastic scattering at 355 nm. We retrieved vertical profiles of WVMRs from the ratio of H_2_O Raman to N_2_ Raman every 20 min^48^. The RL provided vertical WVMR profiles from 200 m asl to 5 km during nighttime and from 0.2 to 1.5 km asl during daytime under cloud-free conditions. The range resolutions were 75 m below 1 km asl and 150 m above 1 km. We eliminated WVMRs with measurement uncertainties exceeding 30% as well as previous studies^35,36^. The estimated accuracy of the WVMR of the RL at the Koshiki site has been evaluated to be 0.88 g kg^−1^ during nighttime^42^. Full details of the RL are available in Yoshida et al.^36^.

The DL observed Doppler velocity every 19 s in five directions: four at a 15° tilt from the zenith and one directly at the zenith. Horizontal wind direction and speed were estimated using the velocity azimuth display technique. The vertical velocity is obtained from the Doppler velocity directed to the zenith. The data acquisition range extended from near the surface to 5 km asl; the vertical resolution was 50 m. The estimated accuracy of the Doppler velocity was less than 0.1 m s^−1^ according to the manufacturer. Several low terrains, mostly less than 100 m asl, were located a few hundred meters west of the Koshiki site. Because westerly winds prevailed around the Koshiki site (Fig. 1d), the terrains triggered gravity waves that might have influenced the DL observations below 600 m asl. To avoid the effects of the gravity waves, we present only the DL observations above 600 m asl.

To overview atmospheric conditions, we used hourly rainfall estimated by weather radar and rain gauge analysis^49^ as well as data via LA that provided hourly meteorological variables at a horizontal interval of 5 km covering all of Japan^43^. The LA data were produced by assimilating various observational data with a three-dimensional variational method. We also employed analysis data of SST at a horizontal interval of 5 km^43^.

## Electronic supplementary material

Below is the link to the electronic supplementary material.

Supplementary Material 1

## Data Availability

The observation data (the RL, DL, and radiosondes) are available from repository (https://doi.org/10.6084/m9.figshare.28099286). The other operational data (LA, RA, weather radar, SST) could be obtained from Japan Meteorological Business Support Centre. Alternatively, the data in this paper can be provided upon request to the corresponding author. For any questions regarding all data, please contact the corresponding author.

## References

[1] Schumacher, R. S. & Rasmussen, K. L. The formation, character and changing nature of mesoscale convective systems. *Nat. Rev. Earth. Environ.* **1**, 300–314 (2020).

[2] Fourrié, N., Nuret, M., Brousseau, P. & Caumont, O. Data assimilation impact studies with the AROME-WMED reanalysis of the first special observation period of the hydrological cycle in the Mediterranean experiment. *Nat. Hazards Earth Syst. Sci.* **21**, 463–480 (2021).

[3] Xu, W. et al. An orography-associated extreme rainfall event during TiMREX: initiation, storm evolution, and maintenance. *Mon. Wea. Rev.* **140**(8), 2555–2574 (2021).

[4] Donat, M., Lowry, A., Alexander, L., O’Gorman, P. A. & Maher, N. More extreme precipitation in the world’s dry and wet regions. *Nat. Clim. Change* **6**, 508–513 (2016).

[5] Prein, A. F. et al. Increased rainfall volume from future convective storms in the US. *Nat. Clim. Change* **7**, 880–884 (2017).

[6] Haberlie, A. M., Ashley, W. S., Gensini, V. A. & Michaelis, A. C. The ratio of mesoscale convective system precipitation to total precipitation increases in future climate change scenarios. *npj Clim. Atmos. Sci.* **6**, 150 (2023).

[7] Huang, Y. et al. Increasing frequency and precipitation intensity of convective storms in the Peruvian Central Andes: Projections from convection-permitting regional climate simulations. *Q. J. R. Meteorol. Soc.* **150**(764), 4371–4390 (2024).

[8] Li, P. et al. Intensification of mesoscale convective systems in the East Asian rainband over the past two decades. *Geophys. Res. Lett.* **50**, e2023GL103595 (2023).

[9] Kato, T. Quasi-stationary band-shaped precipitation systems, named “Senjo-Kousuitai”, causing localized heavy rainfall in Japan. *J. Meteor. Soc. Jpn.* **98**(3), 485–509 (2020).

[10] Peters, J. M., Nielsen, E. R., Parker, M. D., Hitchcock, S. M. & Schumacher, R. S. The impact of low-level moisture errors on model forecasts of an MCS observed during PECAN. *Mon. Wea. Rev.* **145**(9), 3599–3624 (2017).

[11] Schumacher, R. S. Sensitivity of precipitation accumulation in elevated convective systems to small changes in low-level moisture. *J. Atmos. Sci.* **72**(6), 2507–2524 (2015).

[12] Kato, T. et al. Reason for the failure of the simulation of heavy rainfall during X-BAIU-01—importance of a vertical profile of water vapor for numerical simulations. *J. Meteor. Soc. Jpn.* **81**, 993–1013 (2003).

[13] Schumacher, R. S. & Johnson, R. H. Quasi-stationary, extreme-rain-producing convective systems associated with midlevel cyclonic circulations. *Weather Forecasting* **24**, 555–574 (2009).

[14] Trier, S. B., Wilson, J. W., Ahijevych, D. A. & Sobash, R. A. Mesoscale vertical motions near nocturnal convection initiation in PECAN. *Mon. Weather Rev.* **145**, 2919–2941 (2017).

[15] Zhang, M., Meng, Z., Huang, Y. & Wang, D. The mechanism and predictability of an elevated convection initiation event in a weak-lifting environment in Central-Eastern China. *Mon. Weather Rev.* **2019**, 1823–1841 (2019).

[16] Gebauer, J. G., Shapiro, A., Fedorovich, E. & Klein, P. Convection initiation caused by heterogeneous low-level jets over the great plains. *Mon. Weather Rev.* **146**, 2615–2637 (2018).

[17] Schumacher, R. S. & Peter, J. M. Near-surface thermodynamic sensitivities in simulated extreme-rain-producing mesoscale convective systems. *Mon. Weather Rev.* **145**, 2177–2200 (2017).

[18] Crook, N. A. Sensitivity of moist convection forced by boundary layer processes to low-level thermodynamic fields. *Mon. Wea. Rev.* **124**, 1767–1785 (1996).

[19] Zhang, M., Meng, Z., Huang, Y. & Wang, D. The mechanism and predictability of an elevated convection initiation event in a weak-lifting environment in central-eastern China. *Mon. Weather Rev.* **147**, 1823–1841 (2019).

[20] Xu, W. et al. An orography-associated extreme rainfall event during TiMREX: Initiation, storm evolution, and maintenance. *Mon. Weather Rev.* **140**, 2555–2574 (2012).

[21] Jeong, J., Lee, D. & Wang, C. Impact of the cold pool on mesoscale convective system–produced extreme rainfall over southeastern South Korea: 7 July 2009. *Mon. Wea. Rev.* **144**, 3985–4006 (2016).

[22] Parker, M. D. Response of simulated squall lines to low-level cooling. *J. Atmos. Sci.* **65**, 1323–1341. 10.1175/2007JAS2507.1 (2008).

[23] French, A. J. & Parker, M. D. The response of simulated nocturnal convective systems to a developing low-level jet. *J. Atmos. Sci.* **67**, 3384–3408. 10.1175/2010JAS3329.1 (2010).

[24] Kunoki, S. et al. Oceanic influence on the Baiu frontal zone in the East China Sea. *J. Geophys. Res. Atmos.* **120**, 449–463 (2015).

[25] Sato, K. et al. Influence of the Kuroshio on mesoscale convective systems in the baiu frontal zone over the East China Sea. *Mon. Weather Rev.* **144**, 1017–1033 (2016).

[26] Manda, A. et al. Intensive radiosonde observations of environmental conditions on the development of a mesoscale convective system in the Baiu Frontal Zone. *Earth Space Sci.*10.1029/2023EA003486 (2024).

[27] Hirockawa, Y., Kato, T., Tsuguti, H. & Seino, N. Identification and classification of heavy rainfall areas and their characteristic features in Japan. *J. Meteor. Soc. Jpn.* **98**, 835–857 (2020).

[28] Kato, T. Numerical simulation of the band-shaped torrential rain observed over southern Kyushu, Japan on 1 August 1993. *J. Meteor. Soc. Jpn.* **76**, 97–128 (1998).

[29] Kato, T. Structure of the band-shaped precipitation system inducing the heavy rainfall observed over northern Kyushu, Japan on 29 June 1999. *J. Meteor. Soc. Jpn.* **84**, 129–153 (2006).

[30] Ito, J., Tsuguchi, H., Hayashi, S. & Niino, H. Idealized high-resolution simulations of a back-building convective system that causes torrential rain. *J. Atmos. Sci.* **78**, 117–132 (2021).

[31] Nishimura, H. et al. A triggering mechanism of quasi-stationary convective bands in the vicinity of southwestern Japan during the summer season as deduced from moisture origins. *Atmos. Res.* **308**, 107544. 10.1016/j.atmosres.2024.107544 (2024).

[32] Tsuji, H., Takayabu, Y. N., Shibuya, R., Kamahori, H. & Yokoyama, C. The role of free-tropospheric moisture convergence for summertime heavy rainfall in western Japan. *Geophys. Res. Lett.* **48**, e2021GL095030 (2021).

[33] Naka, N., & Takemi, T. Characteristics of the Environmental Conditions for the Occurrence of Recent Extreme Rainfall Events in Northern Kyushu, Japan, SOLA. **19**, 9–16 (2023).

[34] Satoh, M. & Hosotani, K. Characteristics analysis of the Senjo-Kousuitai conditions in the Kyushu region in early July: The Case of the July 2020 heavy rainfall event. *SOLA* **19A**, 1–8 (2022).

[35] Yoshida, S. et al. Lidar observations and data assimilation of low-level moist inflows causing severe local rainfall associated with a mesoscale convective system. *Mon. Weather Rev.* **150**, 1781–1798 (2022).

[36] Yoshida, S. et al. Water vapor lidar observation and data assimilation for a moist low-level jet triggering a mesoscale convective system. *Mon. Weather Rev.* **152**, 1119–1137 (2024).

[37] Kato, R. et al. Improvement of two-hour-ahead QPF using blending technique with spatial maximum filter for tolerating forecast displacement errors and water vapor lidar assimilation. *J. Meteor. Soc. Jpn.* **102**(4), 445–464 (2024).

[38] Schumacher, R. S. & Johnson, R. H. Organization and environmental properties of extreme-rain-producing mesoscale convective systemsm. *Mon. Wea. Rev.* **133**, 961–976 (2005).

[39] Bryan, G. H. & Fritsch, J. M. Moist absolute instability: The sixth static stability state. *Bull. Am. Meteorol. Soc.* **81**, 1207–1230 (2000).

[40] Houze, R. A. Jr. Mesoscale convective systems. *Rev. Geophys.* **42**, RG4003 (2004).

[41] Bauer, H.-S. et al. Evolution of the convective boundary layer in a WRF simulation nested down to 100 m resolution during a cloud-free case of LAFE, 2017 and comparison to observations. *J. Geophys. Res. Atmos.* **128**, e2022JD037212 (2023).

[42] Yoshida, S., Sakai, T., Nagai, T. & Shiraishi, K. Accuracy evaluation of water vapor mixing ratios obtained from water vapor Raman lidars and local analysis data. *J. Laser Radar Soc. Jpn.* **6**(1), 2–13 (2025)*.*

[43] Japan Meteorological Agency, 2019: Outline of the operational numerical weather prediction at the Japan Meteorological Agency. Japan Meteorological Agency, Accessed 20 April 2023, https://www.jma.go.jp/jma/jma-eng/jma-center/nwp/outline2019-nwp/index.htm.

[44] Hirota, N., Takayabu, Y. N., Kato, M. & Arakane, S. Roles of an atmospheric river and a cutoff low in the extreme precipitation event in Hiroshima on 19 August 2014. *Mon. Wea. Rev.* **144**(3), 1145–1160 (2016).

[45] Kato, T. Representative height of the low-level water vapor field for examining the initiation of moist convection leading to heavy rainfall in East Asia. *J. Meteor. Soc. Jpn.* **96**(2), 69–83 (2018).

[46] Ekman, V. W. On the influence of the Earth’s rotation on ocean-currents. *Arkiv Math. Astron. och Fysik* **2**, 1–52 (1905).

[47] O’Brien, J. J. Alternative solutions to the classical vertical velocity problem. *J. Appl. Meteor. Climatol.* **9**, 197–203 (1970).

[48] Sakai, T., Nagai, T., Izumi, T., Yoshida, S. & Shoji, Y. Automated compact mobile Raman lidar for water vapor measurement: Instrument description and validation by comparison with radiosonde, GNSS, and high-resolution objective analysis. *Atmos. Meas. Tech.* **12**, 313–326 (2019).

[49] Nagata, K. Quantitative precipitation estimation and quantitative precipitation forecasting by the Japan Meteorological Agency. *RSMC Tokyo–Typhoon Center Tech. Rev.* **13**, 37–50 (2011).

