# Supplementary material for: Observation of a slanted moisture structure with weak updraft leading to localized heavy rainfalls
Source: Sci Rep. 2025 Jul 2;15:22979. doi: 10.1038/s41598-025-02314-2 (PMC12216889; doi:10.1038/s41598-025-02314-2)
Supplement: Supplementary file 1 — Supplementary Material 1 [file 41598_2025_2314_MOESM1_ESM.docx]

**Supplementary figures**


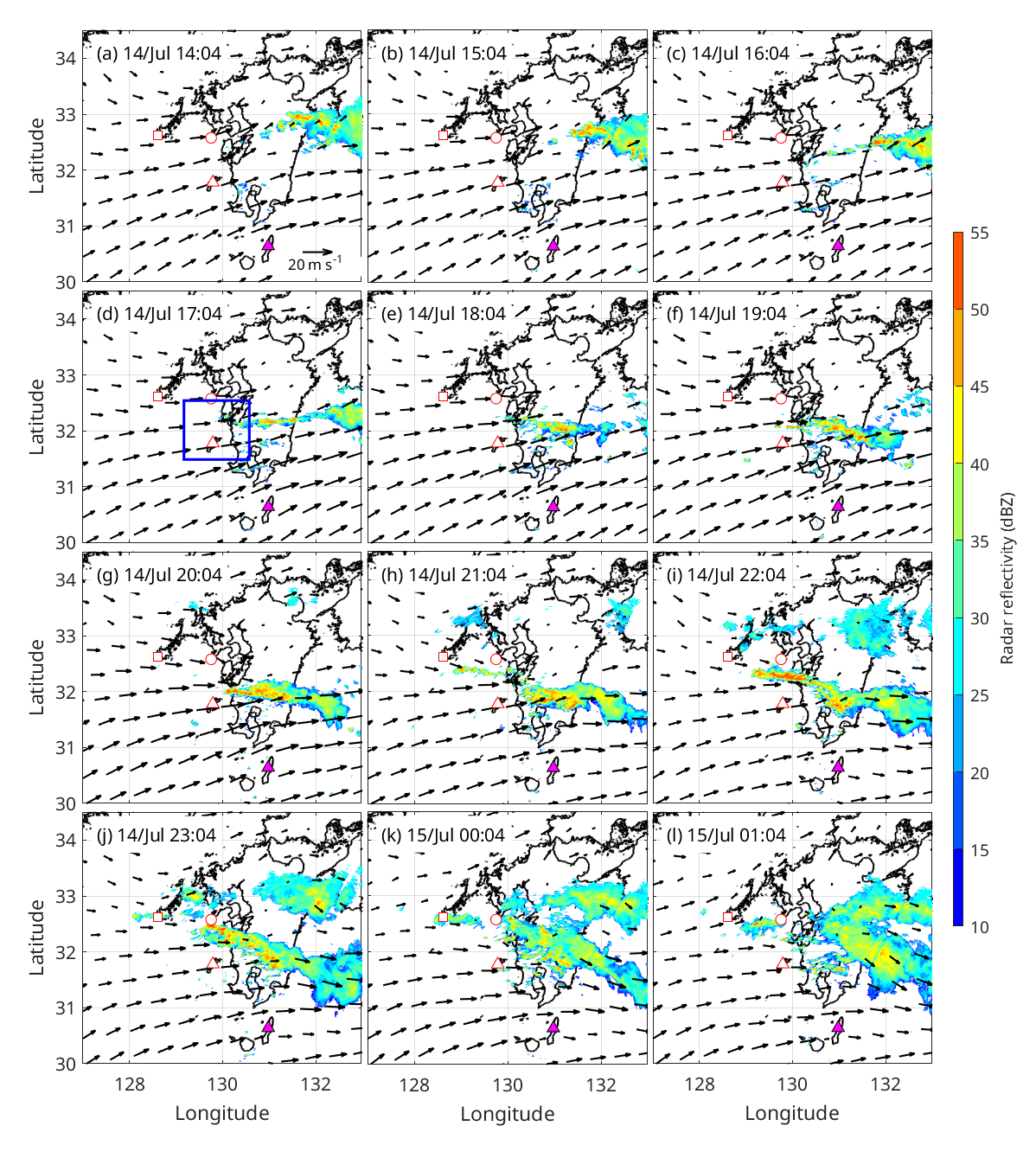


**Fig. S1 | C band Radar reflectivity and horizontal wind fields at 950 hPa level**. The radar reflectivity was shown in plane position indicator with an elevation of 0.3°. The black triangles filled with magenta represent the location of the radar. The horizontal wind fields are from LA. The times in UTC when the radar observations were performed are shown in the upper left corner in each panel. Red squares, circles, and triangles filled with white represent the locations of the Fukue, Nomozaki, and Koshiki sites, respectively. The top-left panel includes a reference arrow that indicates the horizontal wind. A blue rectangle in (d) represent the domain of Fig S2.


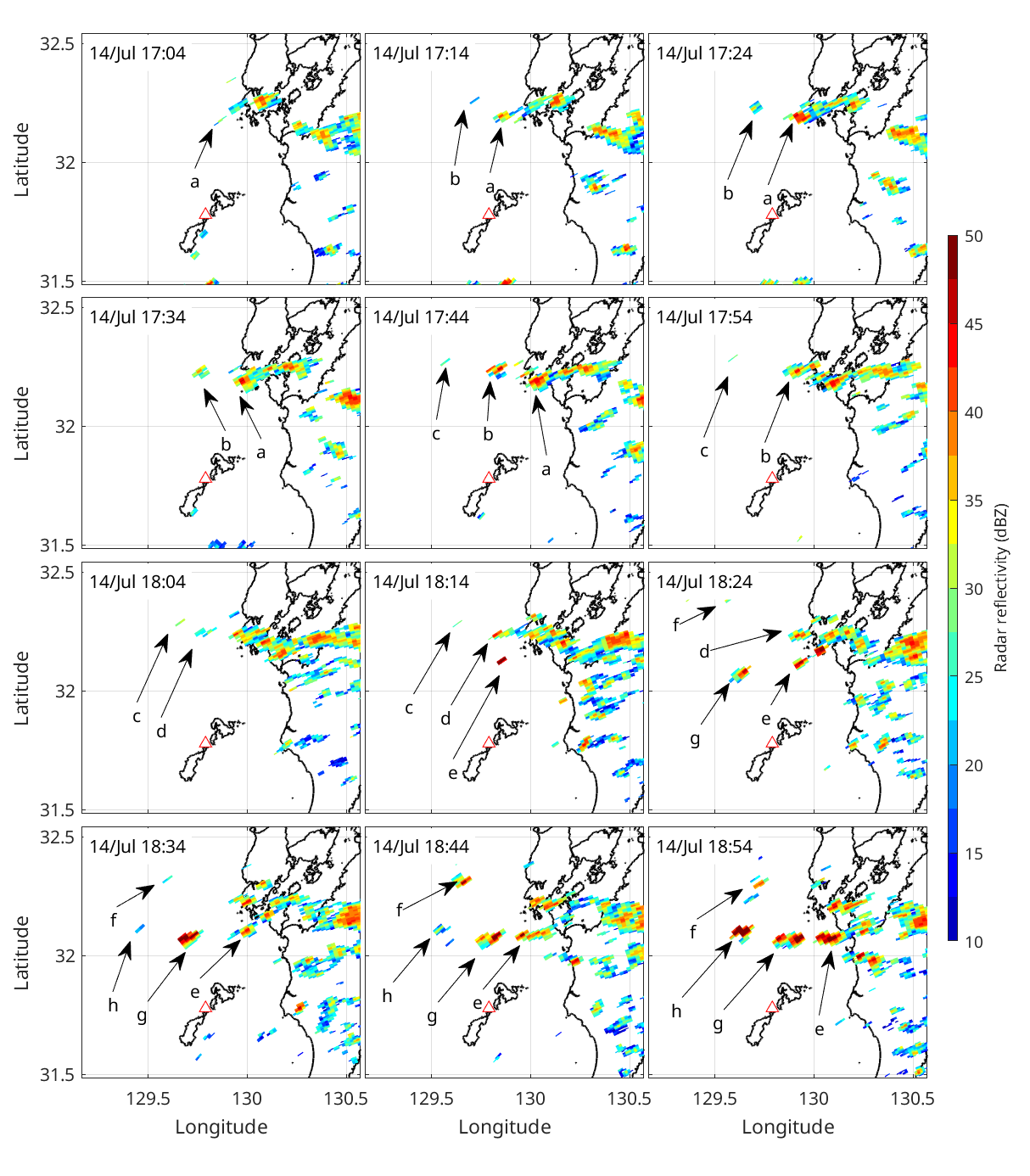


**Fig. S2 | C band Radar reflectivity around Koshiki site every 10 minutes from 17:04 UTC**. The radar reflectivity was shown in plane position indicator with an elevation of 0.05°. The red triangles represent the Koshiki site. The domain of this figure indicated by blue rectangle in Fig. S1d. The radar location of this data is shown in Fig. S1. Each figure indicates convective cells identified by the radar. The convection cells are labeled sequentially from ‘a’ to ‘h’.


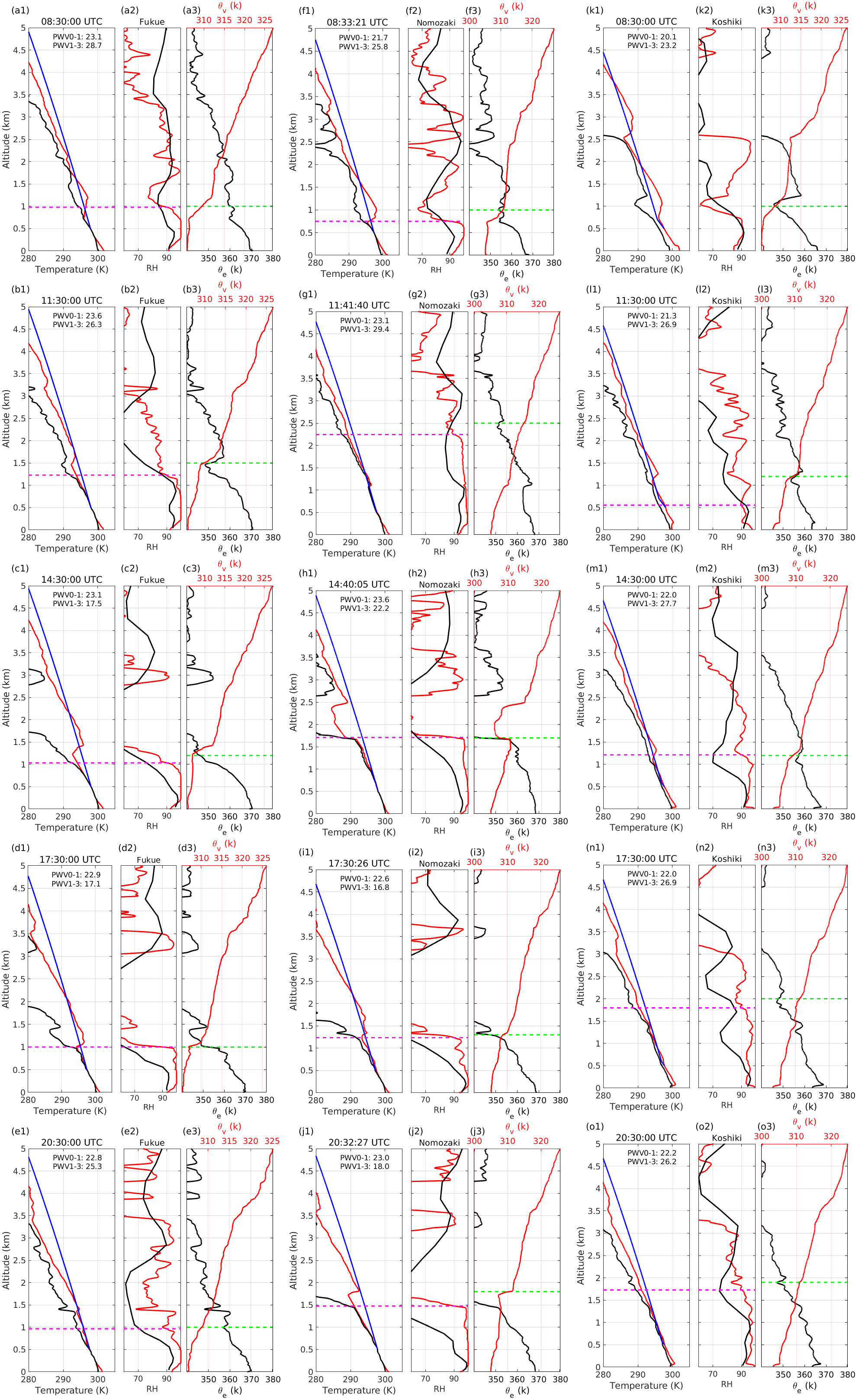


**Fig. S3 | Radiosonde observation results on July 14 2022.** The radiosonde observation results are shown in the same format as Fig.2. Panels in the left column in each group of three represent the results at the Fukue site. Those in the middle column represent the results at the Nomozaki site. Those in the right column represent the results from the Koshiki site. RH90H was not calculated in (k) because of the dry conditions near the surface. Note that (c), (h), and (m) in this figure are identical to (a), (b), and (c) in Fig. 2. See also the caption of Fig. 2.


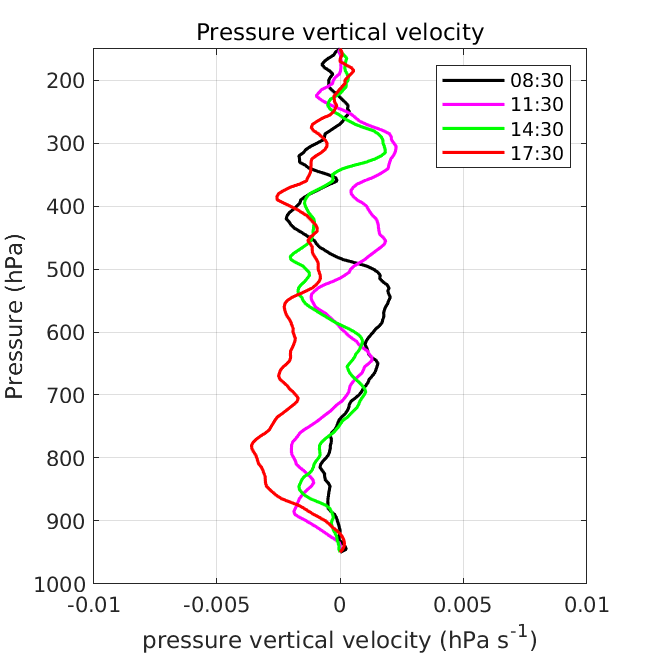


**Fig. S4 | The vertical profiles of vertical pressure velocity on July 14 2022.** The vertical profiles were calculated from the three radiosonde observations approximately at 08:30, 11:30, 14:30 and 17:30 UTC. Note that the time at which the radiosonde was launched is off by as much as 11 minutes.


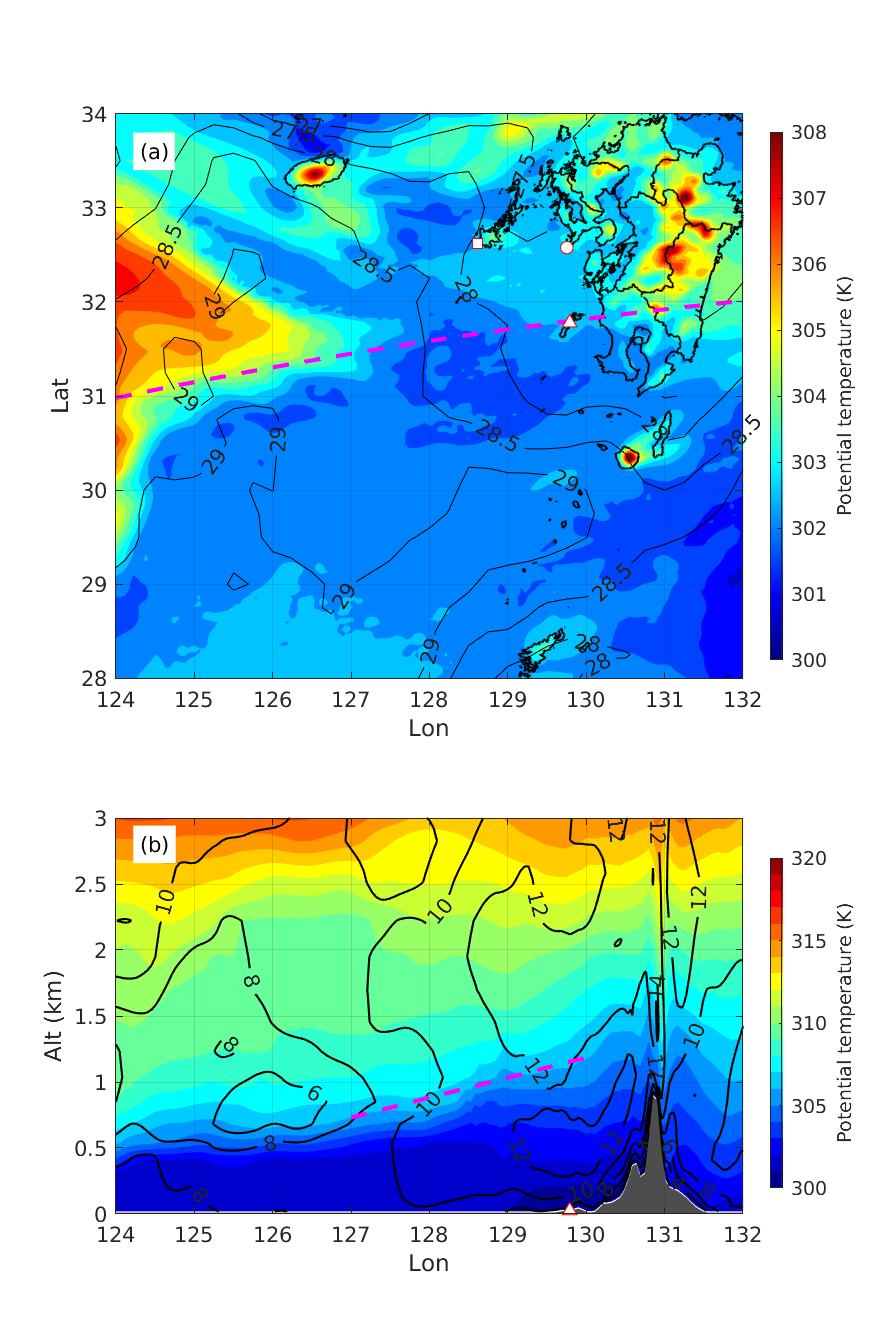


**Fig. S5 | Potential temperature field at 18 UTC on July 14 2022**. (a) horizontal distribution of potential temperature field (color contours) at 950 hPa from LA with SST (black contours with numbers in Celsius) produced in analysis dataset at 18 UTC. (b) Vertical profile of potential temperature (color contours) and horizontal wind speed (black contour lines with numbers) from the LA along the magenta dashed line in (a). The red square, circle, and triangles filled with white represent the location of the Fukue, Nomozaki, and Koshiki sites, respectively. The red triangle and dark gray areas in (b) indicate the location of the Koshiki site and geography. The magenta dashed line in (b) connects the altitude of the 306 K potential temperature at E127° to the altitude of the 306 K potential temperature at E130°.
